# Characterization of the complete mitochondrial genome of *Wuhaniligobius polylepis* (Gobiiformes: Gobiidae) and phylogenetic studies of Gobiiformes

**DOI:** 10.1080/23802359.2018.1519380

**Published:** 2018-10-29

**Authors:** Li Gong, Bingjian Liu, Zhen-Ming Lü, Li-Qin Liu

**Affiliations:** a National engineering Laboratory of Marine Germplasm Resources Exploration and Utilization, Zhejiang Ocean University, Zhoushan, China;; b National engineering research center for facilitated marine aquaculture, Marine science and technology college, Zhejiang Ocean University, Zhoushan, China

**Keywords:** Wuhaniligobius polylepis, complete mitogenome, phylogenetic analyses

## Abstract

The environmental changes mainly caused by water pollution and human activities have dramatically threatened the survival of the small-scaled Wu’s goby *W. polylepis*. In the present study, the complete mitogenome of *Wuhaniligobius polylepis* was determined, which is 16,496 bp in length, containing 13 PCGs, two rRNA genes, 22 tRNA genes and a putative control region. The overall base composition is 28.6%, 29.0%, 26.7%, and 15.7% for A, T, C and G, respectively. The 13 PCGs encode 3,802 amino acids in total, twelve of which use the initiation codon ATG except *COI*, which uses GTG. In order to explore the systematic status of *W. polylepis* and further phylogenetic study of Gobiiformes, a maximum-likelihood tree was constructed based on the concatenated sequences of 12 PCGs. The result showed that *Pseudogobius*, *Hemigobius,* and *Mugilogobius* clustered into a clade and then formed a sister-group with *W. polylepis*, suggesting the invalid former name of *M. polylepis* or *E. polylepis*.

The small-scaled Wu’s goby *Wuhaniligobius polylepis* (Wu and Ni 1985), formerly called *Mugilogobius polylepis* or *Eugnathogobius polylepis*, distributed in eastern and southern China is found in mangroves and coastal habitats (Nelson 2006). The environmental changes caused by water pollution and overfishing, have dramatically threatened the survival of *W. polylepis* (Huang et al. 2013). So far, there are limited molecular studies of this Endangered species, which greatly hinders the phylogenetic studies and further conservation strategies. In order to provide useful molecular information for the restoration strategies and phylogenetic studies for this species, we determined and described the complete mitochondrial genome of *W. polylepis* and explored the phylogenetic relationship among all the Gobiiformes species, aiming for the phylogenetic studies of Gobiiformes and further management for this species.

The specimen was collected from Beihai, China (N21°29′20″, E109°07′12″) and stored in a refrigerator at −80 °C with accession number 20171016BH02. The total length is 16,496 bp (GenBank No. MG744345). The gene arrangement is identical to that of the typical vertebrate mtDNA (Boore 1999; Gong et al. 2017a; Gong et al. 2017b), including 13 protein-coding genes (PCGs), two rRNA genes, 22 tRNA genes, and a putative control region. Among the 37 genes, nine genes were encoded on the light strand, and the remaining 28 genes were encoded on the heavy strand. The overall base composition is 28.6%, 29.0%, 26.7%, and 15.7% for A, T, C, and G, respectively. The 13 protein-coding genes encode 3,802 amino acids in total. The most frequently used amino acid is Leucine (16.23%), while Cysteine acid (0.76%) is the least frequently used one. Twelve of the PCGs use the initiation codon ATG except for *COI,* which uses GTG. Seven of them (*ND1*, *ND2*, *COI*, *ATP8*, *ATP6*, *ND4L* and *ND5*) have TAA as the stop codon, while *ND4* is inferred to termination with AGG, *ND3* and *ND6* with TAG, and the remaining four (*COII*, *COIII*, and *Cyt b*) are inferred to termination with an incomplete stop codon T.

The major non-coding sequence or control region (CR) is commonly located between the *tRNA-Pro* and *tRNA-Phe* genes. It is 839 bp in length and is characterized by a relatively high AT content (65.4%). The base composition is 32.1%, 33.3%, 19.2%, and 15.5% for A, T, C, and G, respectively. Like that of other vertebrates (Guo et al. 2003; Ragauskas et al. 2014; Wang et al. 2014; Gong et al. 2015), the CR of this species is also partitioned into three domains and some similar conserved-sequence blocks are found, including the termination associated sequence (TAS), the central conserved sequence blocks (CSB-F, D, C and A) and the conserved sequence blocks (CSB-2 and 3).

In order to explore the systematic status of *W. polylepis* and carry out further phylogenetic study of Gobiiformes, a maximum-likelihood tree was constructed based on the concatenated sequences of 12 PCGs. The tree clearly showed that *Pseudogobius*, *Hemigobius,* and *Mugilogobius* are clustered into a clade and form a sister-group with *W. polylepis*, suggesting that the former name of *M. polylepis* or *E. polylepis* (Figure 1) was invalid.

**Figure 1.**
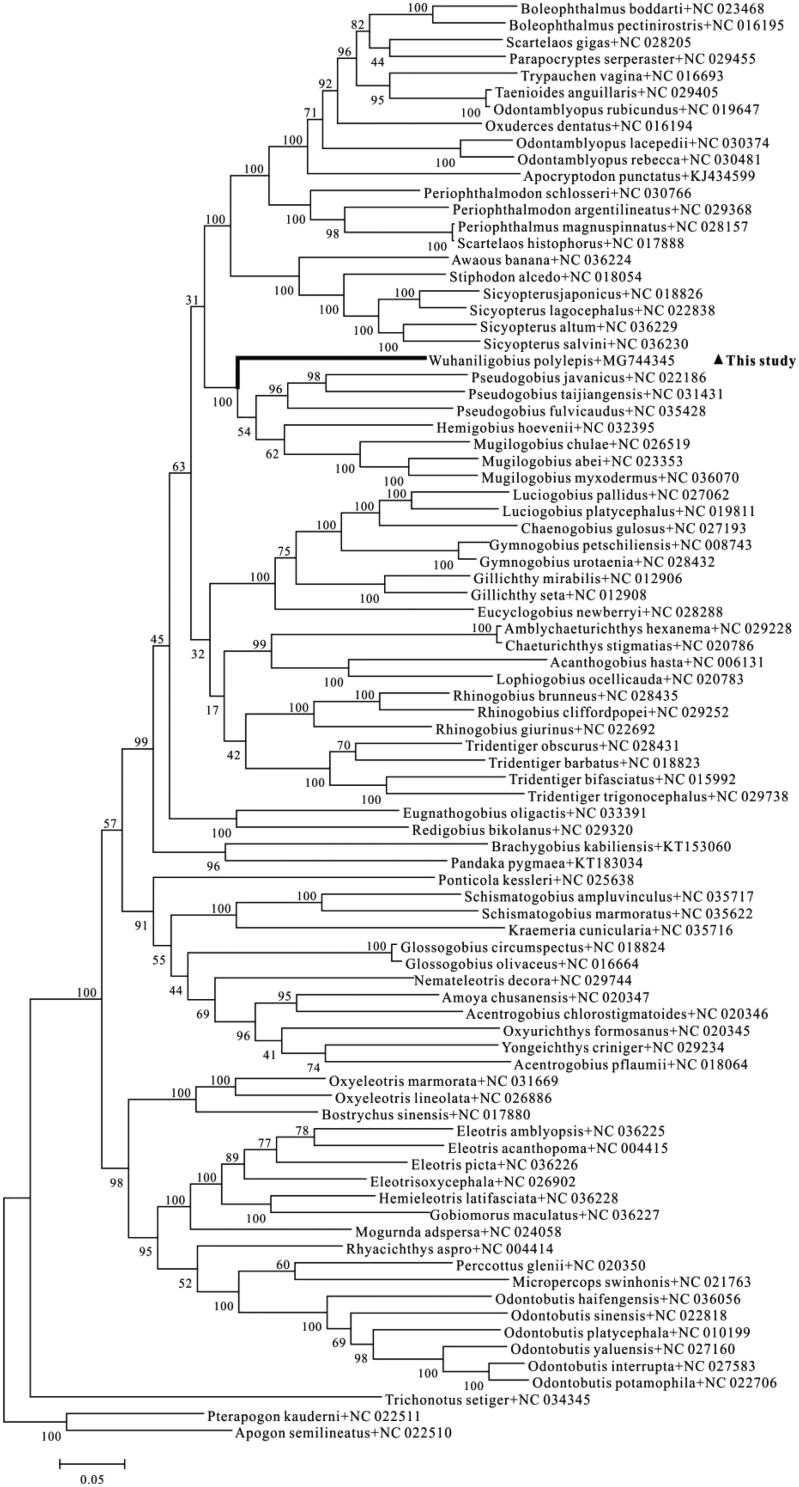
Maximum Likelihood (ML) tree of Gobiiformes species based on 12 PCGs. The bootstrap values are based on 500 resamplings. The number at each node is the bootstrap probability. The number after the species name is the GenBank accession number. The genome sequence in this study is labeled with triangle.
